# A physician-pharmacist partnership intervention for deprescribing (P3iD) among older adults attending a falls and syncope clinic: Protocol for a randomised controlled trial

**DOI:** 10.1371/journal.pone.0324565

**Published:** 2025-06-03

**Authors:** Sheron Sir Loon Goh, Pauline Siew Mei Lai, Siti Nurkamilla Ramdzan, Kit Mun Tan, Amanda Mae Ching Goh, Maw Pin Tan

**Affiliations:** 1 Department of Clinical Pharmacy and Pharmacy Practice, Faculty of Pharmacy, Universiti Malaya, Kuala Lumpur, Malaysia; 2 Department of Primary Care Medicine, Faculty of Medicine, Universiti Malaya, Kuala Lumpur, Malaysia; 3 School of Medical and Life Sciences, Sunway University, Sunway, Selangor, Malaysia; 4 Division of Geriatric Medicine, Department of Medicine, Faculty of Medicine, Universiti Malaya, Kuala Lumpur, Malaysia; Hospital Sirio-Libanes, BRAZIL

## Abstract

**Background:**

The concept of deprescribing is gaining traction among clinicians as a formalized approach to improving medication safety for older persons. It has been found to be safe and effective in reducing medication burden. However, its implementation remains challenging. Most research has been conducted in high-income countries, with limited prospective data on deprescribing outcomes in outpatient care settings for older adults in low- and middle-income countries (LMICs). Therefore, evaluating local deprescribing interventions is essential to produce evidence on their effectiveness in older populations. Our study aimed to assess the effectiveness of the Physician-Pharmacist Partnership Intervention for Deprescribing (P3ID) among older persons attending a falls and syncope clinic.

**Methods:**

This randomised controlled trial will be conducted at a teaching hospital in Kuala Lumpur, Malaysia. Participants will involve individuals aged ≥60 years with at least one chronic disease requiring pharmacological treatment, attending the falls and syncope clinic with ≥1 potentially inappropriate medication (PIM) undergoing systematic multidomain assessment and attending physicians at the clinic. The joint pharmacist-physician intervention comprises five steps: 1) PIM identification, 2) decision on cessation and prioritisation, 3) medication withdrawal, 4) monitoring and support, 5) and documentation.

**Conclusion:**

The P3ID trial tests the hypothesis that a jointly led pharmacist-physician intervention in an outpatient will reduce the total number of medications, improve medication adherence, reduce falls and improve patients’ and doctors’ satisfaction towards pharmacist services. Findings from this study would inform future deprescribing practices, particularly in LMIC, pertaining to fall prevention as well as aid the development of future deprescribing interventions in other settings.

## Introduction

Deprescribing is defined as “the process of withdrawal of an inappropriate medication, supervised by a healthcare professional with a goal of managing polypharmacy and improving outcomes” [1]. One in five medications prescribed to older persons are considered inappropriate [2]. In Malaysia, 13.6% of older persons attending an outpatient clinic have been prescribed at least one potentially inappropriate medication (PIM) [3]. PIMs are associated with an increased risk of adverse drug events, increased healthcare utilisation, prolonged hospitalization and increased mortality, especially in older persons with multiple chronic conditions [4].

The recognition of harms caused by PIMs has led to research and clinical practice interventions targeted at deprescribing PIMs [1] Several deprescribing tools and interventions, such as medication reviews by pharmacists, PIM identification tools, and patient education materials are available to aid deprescribing in clinical practice [5–7]. A previous systematic review found that pharmacist-led deprescribing interventions were effective in reducing PIM use and medication burden for older ambulatory adults [8]. Pharmacists are valuable collaborators in deprescribing and are capable of leading deprescribing interventions by providing necessary monitoring throughout the tapering and monitoring processes [9].

The concept of deprescribing concept is also now increasingly popular among clinicians as a formalised approach to improving medication safety for older persons. Based on a feasibility study conducted using a physician-pharmacist partnership intervention developed in a primary care setting, deprescribing was successfully performed in 53% of PIMs found in older persons attending the clinic [10]. A systematic review found that deprescribing was safe and reduced number of medications as well as improved health-related quality of life (HRQOL), reduced healthcare costs, and hospitalization [11]. However, despite these promising outcomes, the review also highlights a critical gap: most of the evidence comes from high-income countries, with limited data available from low- and middle-income countries (LMICs). Furthermore, the lack of prospective outcome data on deprescribing in outpatient settings—where the majority of older persons are managed—remains a significant barrier to widespread implementation, particularly in resource-limited settings. This underscores the importance of evaluating deprescribing interventions in LMICs. By generating local evidence on its effectiveness in improving clinical outcomes and reducing medication burden in older adults, particularly in the falls and syncope clinic, this study will provide critical insights into how deprescribing can be optimized and scaled in LMIC contexts. Hence, our study aims to determine the effect of a Pharmacist-Physician Partnership Intervention for Deprescribing (P3ID) on reducing the total number of prescribed medications among older adults attending a falls and syncope clinic. In addition, the secondary outcomes of medication adherence, fall occurrence, rate of falls and patients’ and doctors’ satisfaction will also be evaluated.

## Materials and methods

### Study design and setting

A randomised controlled trial will be conducted at a teaching hospital. This hospital serves as a general hospital for around 300,000 people in its immediate catchment and provides tertiary services to public and private hospitals nationwide. The falls and syncope clinic receives referrals from various sources, including the emergency department, general practitioners, primary care doctors, specialist outpatient clinics, and hospital wards, both from within the hospital and from other community and hospital settings [12]. All patients referred to the clinic will be classified as high risk for falls and will undergo a multidomain risk assessment, including a medication review, in accordance with the World Guidelines for Falls Prevention and Management in Older Adults [13].

### Participants

There will be two main groups of participants in this study: patients and physicians.

#### Patients.

Individuals aged ≥60 years with at least one chronic disease requiring pharmacological treatment attending the falls and syncope clinic with ≥1 PIM according to the STOPPFall criteria [14] undergoing systematic multidomain assessment will be included. Researchers and their attending physician assessed participants’ capacity to provide informed consent for this study based on clinical judgment and provided training on mental capacity assessments. Written informed consent is obtained from participants who are deemed to have capacity. For those who lack mental capacity, assent is obtained from their next of kin. For those who may lack mental capacity, assent will be obtained from their next of kin. Since medication review and deprescribing are generally considered appropriate for older adults, no exclusion criteria are anticipated. However, consent will be sought from the physician overseeing the participant’s care, who will also be recruited into the study. If the physician determines that participation in the RCT is unsuitable for the participant based on clinical judgment, the participant will be excluded. We acknowledge the challenges of including older adults with cognitive impairment in falls prevention studies, but believe it is important for real-world relevance; while past interventions like Shaw et al. 2003 [15] focused on multifactorial strategies, deprescribing studies have shown feasibility and safety in this population with proper safeguards [16–18]. Although inclusion may attenuate the intervention effect, our approach prioritises external validity, with caregiver support and adapted data collection methods ensuring reliable participation and outcomes [19,20]. Additionally, if a potential participant’s physician declines to participate and the participant chooses to remain under their care, the participant will also be excluded.

#### Physicians.

Geriatricians, internal and family physicians undergoing subspecialty training in geriatric medicine and medical officers working at the centre will be recruited.

### Sample size calculation

Sample size was calculated using the Open Source Epidemiologic Statistics for Public Health (OpenEpi version 3.01) [21]. A search of published literature found that the mean change in the number of prescribed medications at 12 months was −1.9 ± 4.1 in intervention group participants and +0.1 ± 3.5 in control group participants [22]. Hence, the minimum number of participants needed with a two-sided confidence level of 95%, and 80% power will be at least 58 participants in each group [21]. Assuming a 20% attrition rate [23], the total number of 70 participants will be recruited to each arm.

### Procedure

**Fig 1:**
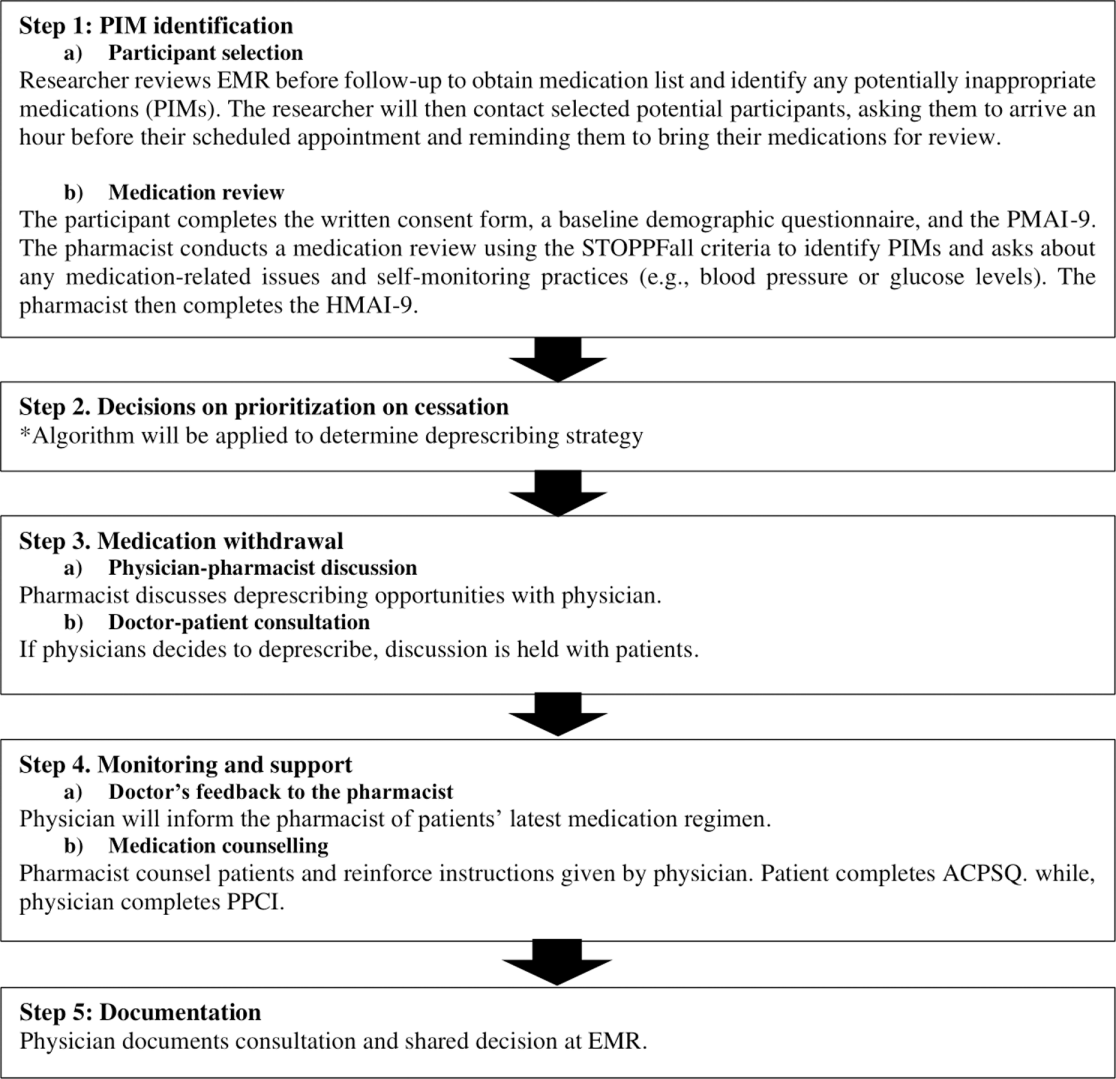
Flowchart on the five-steps of the physical-pharmacy partnership in deprescribing intervention.

The researcher will first approach physicians attending to potential participants individually to explain the study workflow using the participant information sheet and their involvement in the deprescribing process for the intervention group. Written informed consent will be obtained from the physicians. Consenting physicians will be asked to complete the baseline demographic form Fig 1.

**Fig 2:**
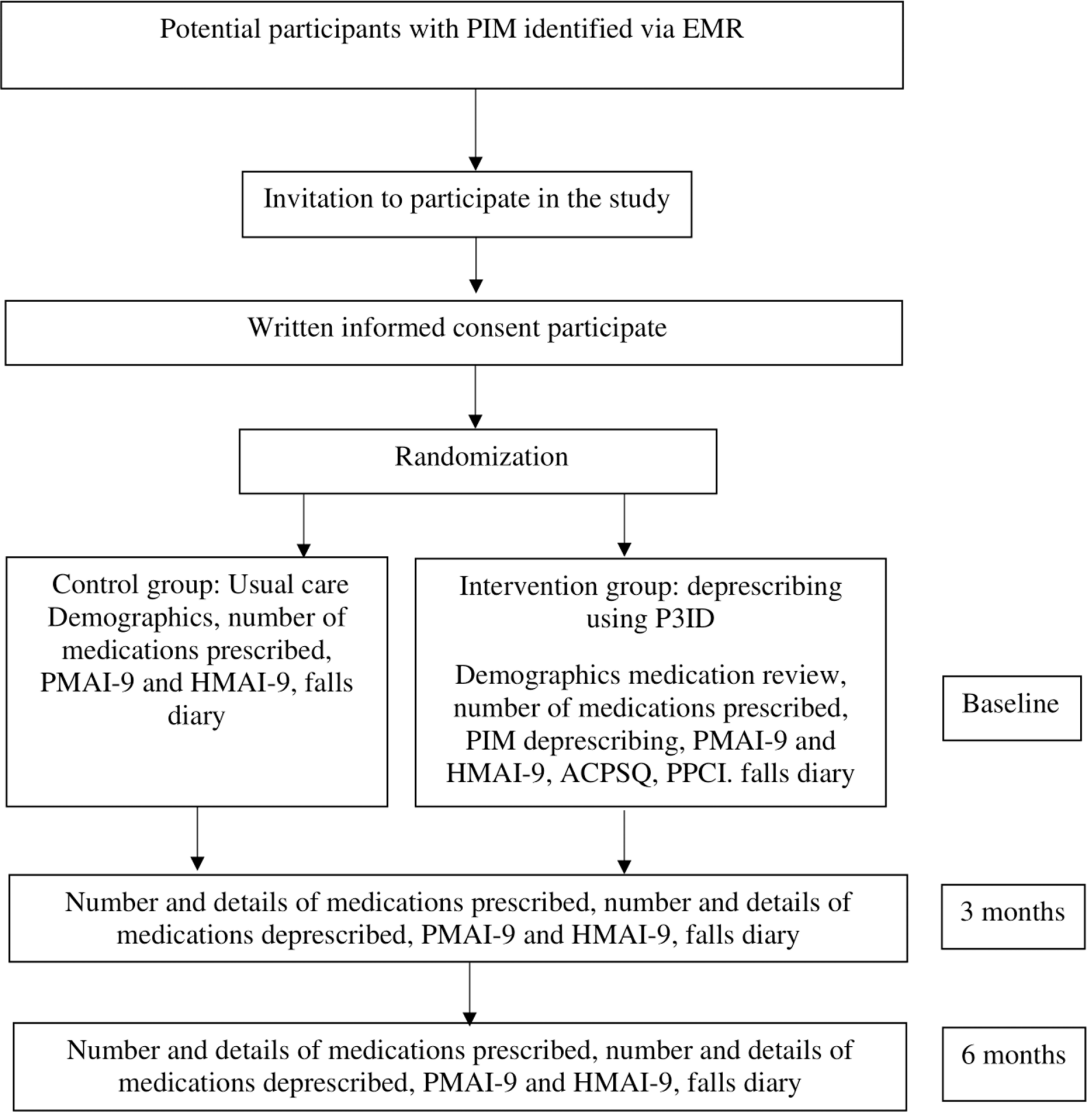
Consort flow diagram on participant recruitment and follow-up .

The researcher will review potential participant’s electronic medical records (EMR), if available, on the day before their appointment. The researcher will first approach the physician attending to the potential participant to determine suitability for the study before approaching selected potential patients to explain the purpose of the study. If the individual agrees to participate in the study, they will be randomised to an intervention (deprescribing) or control group (usual care) in a 1:1 ratio using a computerized random number generator in Microsoft Office® Excel® (Washington, United States of America) [24]. Participants randomized to the control group will continue to receive usual care, where medications will be reviewed and prescribed by their physician. Meanwhile, patients in the intervention group will receive an in-depth medication review performed by a pharmacist, which will focus on identifying PIMs as opportunities for deprescribing. In the intervention arm, a registered pharmacist will perform medication reconciliation using a structured medication list. However, in the control group, physicians will rely on electronic medical records and routine medication history-taking. PIMs identified in the intervention group will be deprescribed using the P3ID intervention, as shown in Participants will be followed up over 6 months (Fig 2). To assess the primary outcome, the number of prescribed medications will be determined by reviewing the patient’s medications through EMR and patient interviews via telephone calls at 3 months and 6 months. Medication adherence will be assessed using PMAI-9 and HMAI-9 via telephone calls at 3 months and 6 months. All participants will be requested to record any adverse drug reactions (ADR) due to their prescribed medications. For those undergoing deprescribing, they will be informed about the potential of withdrawal-related adverse effects, such as the return of previous symptoms or other withdrawal reactions. They will be asked to monitor and record any such symptoms. and asked to record these symptoms or withdrawal reactions. They will also be advised to see a doctor if they feel unwell or notice any unexpected adverse effects after stopping the medication.

A monthly falls diary with daily entries will be used to record fall occurrences which will returned through conventional mail for 6 months from randomization. When participants will be contacted at 3 and 6 months by telephone to encourage complete diary returns, at the same time they will also be asked to report the presence of any fall in the preceding three months. The date of falls will also be established, and if the participant has problems recalling the exact date, the week or month of falls will be recorded.

### Outcome measures

The primary outcome of this study will be the total number of prescribed medications. Secondary outcomes measured will include the number of medications deprescribed, fall occurrence, falls rate, PMAI-9 and HMAI-9. Within the intervention arm, additional information will be obtained on PIM, number of PIM deprescribed, and patients’ and doctors’ satisfaction towards pharmacist services.

### Instruments used

The instruments used for this study are described in detail in Table 1. Each instrument is outlined based on its purpose and the specific data it collects.

**Table 1:** Instruments used and timepoints.

No	Instruments	Description	Instruments to be completed by	Time-points
1	Baseline demographic questionnaire	to collect doctors’ demographic data	Physicians, patients	Baseline
3	STOPPFall	to analyse patients’ medication list for any PIMs.	Pharmacist	Baseline
4	Patient-Medication Adherence Instrument (PMAI-9)	to assess patients’ adherence towards their medications from the patient’s perspectives	Patients	Baseline, 3 month, 6 month
5	Healthcare-Professional-Medication Adherence Instrument (HMAI-9)	to assess patients’ adherence towards their medications from and HCP’s perspectives	Pharmacist	Baseline, 3 month, 6 month
6	Patient assessment form	to document the medical history, medication history, falls history, syncope symptoms and its precipitating factors, clinical frailty scale score and deprescribing intervention in older persons.	Pharmacist	Baseline, 3 month, 6 month
7	Ambulatory Care Patient Satisfaction Questionnaire (ACPSQ)	to assess quality of pharmacy services delivered by the pharmacist.	Patients	Baseline
8	Physician–Pharmacist Collaborative Index (PPCI)	to assess the professional exchanges between doctors and the pharmacist.	Physicians	Baseline
9	Falls diary	to record any falls occurrence in older persons. Prompts will be provided in the falls diaries, which include how and when a fall occurred, or if there were injuries sustained after their fall.	Patients	Daily (Diary returns at 3 month and 6 month)

### Data analysis

Data will be analysed using the Statistical Package for the Social Sciences (SPSS) version 21 (IBM^TM^, Armonk, New York, United States). A normality test will be performed. Descriptive statistics will be presented as percentages and frequencies, whilst continuous variables will be calculated using means and standard deviation. Raw, unadjusted, outcomes data will be presented. No hypothesis testing will be conducted for baseline variables given that any differences would have occurred at random. For comparisons of subsequent follow-up data, categorical variables will be analysed using the chi-squared test while the independent t-test will be used for continuous variables.

Outcomes analysis will be conducted on an intention-to-treat basis. All outcome variables that contain less than 10% missing data, will be considered missing at random and no replacement will be conducted. For outcome variables with greater than 10% missing data, missing data analyses will be conducted. Data missing not at random will be replaced using multiple imputations. The primary outcome measurement of the total number of medications per person will represent a continuous variable. Between-group and within-group analyses will be conducted using repeated measures analysis of variance. The remaining outcome variables will be handled according to whether each variable is categorical or continuous, normally or non-normality distributed, and the number of observations obtained. Variables with single measurements will be compared using logistics or linear regression analysis while those with multiple measurements with repeated measures analysis of variance or general estimating equations. Unadjusted analyses will first be presented. Potential confounders will be selected using available published evidence and clinical judgement.

### Ethics approval

Ethics approval was obtained from the local institutional review board (MREC ID NO: 2023630−12610).

## Discussion

This study is intended to address the evidence gap on deprescribing in Asian healthcare settings, particularly in LMIC within Asia [25,26]. Unlike previous research, which has mainly been conducted in North America, Western Europe, and Australasia among predominantly white Caucasian populations [27,28], the findings may not be fully applicable to Asian LMICs due to differences in culture, healthcare resources, and system structures [29]. Our deprescribing intervention is part of a multidomain approach, which combines physician and pharmacist collaboration for a more comprehensive strategy. This multifactorial method is expected to enhance benefits, such as reducing PIMs, adverse drug reactions, and falls, ultimately improving patient outcomes and lowering healthcare costs.

This study is intended to address the evidence gap on deprescribing in Asian healthcare settings, particularly in LMIC within Asia [26]. While, previous research has shown that deprescribing interventions effectively reduce PIMs, adverse drug reactions, and falls, improving patient quality of life and reducing healthcare costs, these studies have primarily been conducted in white Caucasian countries within North America, Western Europe, and Australasia [27,28]. Available evidence, therefore, potentially lacks applicability within Asian LMIC given the differences in culture, resources and healthcare systems [29].

Medication management in older persons remains a challenge in Malaysia. Both patients and doctors acknowledge the necessity of deprescribing but are hesitant due to concerns about disrupting established treatment plans [30]. Similarly, despite pharmacists’ crucial role in deprescribing, [31], the implementation of this role is still in its infancy, particularly in outpatient geriatric care. This may be due to a hierarchical structure within the healthcare system, where doctors often hold greater authority over clinical decisions, including prescribing practices [32]. In geriatric care, pharmacists’ roles must be expanded and better integrated into patient care teams including working closely with doctors to regularly assess medication regimens. Collaborative efforts between pharmacists and other healthcare providers are necessary to ensure safe and effective deprescribing practices [33].

Recruitment of participants at a specialist clinic from a single study site limits the generalizability of our results to wider secondary care settings in Malaysia and elsewhere. The results are primarily applicable to settings where multidomain falls risk assessment is already implemented in accordance with international guidelines. While many patients in falls clinics meet the inclusion criteria for deprescribing, managing these patients and determining the most appropriate medications to deprescribe can be complex and require careful prioritisation to avoid potential harm related to omission of crucial treatment drugs. Medications that affect balance or cognition such as sedatives or antihypertensives can be difficult to deprescribe because of withdrawal symptoms or perceived necessity for managing chronic diseases.

Withdrawal of medications for chronic conditions, such as blood pressure and glucose lowering agents, proton pump inhibitors and antipsychotics, may negatively affect disease or symptom control leading to the medication being restarted [34]. The successful implementation of the deprescribing process, therefore, does require systemic changes through multiple levels of healthcare practice [35].

Deprescribing often requires close collaboration within the multidisciplinary team. Therefore, this RCT will allow us to evaluate the effectiveness of the deprescribing intervention in a smaller, controlled setting with a limited group of healthcare professionals in a highly poly-pharmaceutical patient population in which adverse outcomes have already occurred and further adverse outcomes are likely. However, expanding the intervention to a larger scale can face challenges, including the need for broader acceptance from prescribers, standardized protocols, and better coordination of care, as fragmented communication between providers. Cost-effective evaluation would be invaluable to inform policy, and should be included in future studies.

### Clinical implications

A step-by-step approach on how to deprescribe medications in older persons in outpatient care has been developed to guide clinicians through the process of reducing medication burden and in turn address potential medication-related harm. The findings from this study will provide valuable insights for the implementation of deprescribing in real-world clinical practice which will inform the development of deprescribing guidelines and contribute to the Malaysian National Medicines Policy (DUNAS). The study will also have a positive impact on the pharmacy profession by expanding the pharmacist’s role in clinical practice with the desired benefit of improved patient outcomes and reduction in medication errors. Further, the findings of this study could also inform deprescribing practices in other LMICs and resource-limited settings within high-income countries.

## Conclusion

This paper describes the protocol of a randomised controlled trial to evaluate a deprescribing intervention jointly led by physicians and pharmacists, conducted on individuals attending a specialist falls clinic, with the primary outcome of total number of prescribed medications. This study is much needed as it addresses the gap in evidence in interventions for deprescribing among older adults in LMIC and Asia. The findings of this study will potentially lead to larger, multi-centre studies on deprescribing within these settings for falls prevention and other outcomes which matter to the older adult. The study will also have important implications for policies and clinical practice in the region.
